# Improvement of the antibody-dependent respiratory burst assay for assessing protective immune responses to malaria

**DOI:** 10.1098/rsob.210288

**Published:** 2022-03-16

**Authors:** Annick Mansourou, Charlotte Joos, Oumy Niass, Babacar Diouf, Adama Tall, Ronald Perraut, Makhtar Niang, Aissatou Toure-Balde

**Affiliations:** ^1^ Unité Immunologie, 36 Avenue Pasteur, 220 Dakar, Sénégal; ^2^ Unité Epidémiologie, 36 Avenue Pasteur, 220 Dakar, Sénégal

**Keywords:** malaria, *Plasmodium falciparum*, merozoites, functional assay, neutrophils, respiratory burst

## Abstract

The antibody-dependent respiratory burst (ADRB) assay is a sensitive isoluminol-based chemiluminescence (CL) functional assay designed to assess the capacity of opsonizing antibodies against merozoites to induce neutrophil respiratory burst. ADRB was shown to measure protective immunity against malaria in endemic areas, but the assay needed further improvement to ensure better sensitivity and reproducibility. Here, we adjusted parameters such as the freezing–thawing procedure of merozoites, merozoites's concentration and the buffer solution's pH, and we used the improved assay to measure ADRB activity of 207 sera from 97 and 110 individuals living, respectively, in Dielmo and Ndiop villages with differing malaria endemicity. The improvement led to increased CL intensity and assay sensitivity, and a higher reproducibility. In both areas, ADRB activity correlated with malaria endemicity and individual's age discriminated groups with and without clinical malaria episodes, and significantly correlated with *in vivo* clinical protection from *Plasmodium falciparum* malaria. Our results demonstrate that the improved ADRB assay can be valuably used to assess acquired immunity during monitoring by control programmes and/or clinical trials.

## Graphical abstract

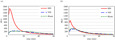

## 1. Background

Malaria due to *Plasmodium falciparum* still remains an important public health concern despite the dramatic decline of malaria incidence recorded over the past fifteen years, and to which malaria control measures such as insecticide-treated bed nets, indoor residual sprays of insecticides, widespread use of rapid diagnostic test and artemisinin combination therapies have collectively contributed significantly [1]. According to the World Malaria Report 2020, there were 229 million cases and 409 000 deaths globally attributed to malaria in 2019 [1].

To date, vaccination is widely acknowledged as the ultimate strategy to eliminate malaria. However, efforts to develop and validate an effective vaccine against malaria infection or transmission face many challenges, such as the lack of validated correlates of protective immunity against malaria. Functional assays have the potential to reliably identify the key protective antigens to be included in a safe and effective vaccine against malaria [2].

Merozoites—the extracellular forms of the parasite—play a critical role in the invasion of the erythrocytes and are exposed to antibodies and immune cells in the bloodstream. Merozoite surface-associated antigens thus represent major targets for blood-stage vaccine development [2,3]. Specific cytophilic antibodies of IgG1 and IgG3 isotypes, rather than the absolute levels of IgG responses to merozoite antigens, were associated with protective immunity in individuals naturally exposed to malaria, as demonstrated by several immuno-epidemiological studies [4–6]. To date, it is still unclear how and which of the many merozoite specific-antibodies are responsible for a protective effect *in vivo*, but it is thought that functionally associated protection occurs through various mechanisms, such as inhibition of merozoite invasion into erythrocytes [7], cooperation between effector cells and merozoite-opsonizing antibodies binding to specific receptors for cytophilic IgG [Fcγ-receptors (Fc*γ*Rs)] expressed on the cells surface of phagocytes to trigger anti-parasitic effect such as merozoite phagocytosis [8–10], or the release of soluble factors responsible for the destruction of merozoites [9,11–13].

Actually, none of the functional assays commonly used for malaria vaccine evaluation, such as the growth inhibition assay [14,15] or the antibody-dependent cellular inhibition of parasite growth [12], are validated as a reliable predictor of immune status *in vivo* in prospective longitudinal studies. Therefore, the development of new reliable and reproducible functional assays to identify and measure protection-associated mechanisms against clinical malaria remains an urgent need among the malaria research priorities [16,17]. In recent years, there has been a renewed interest in the development of *in vitro* functional assays based on the protective effect of IgG opsonic antibodies Fc-dependent mediating anti-merozoite activities [18–21].

In light of this, a high-throughput isoluminol-based chemiluminescence (CL) assay called the antibody-dependent respiratory burst (ADRB) assay has been described [22]. The ADRB assay measures the functional ability of opsonizing antibodies against merozoite to interact with human neutrophils via their Fc*γ*Rs to induce a respiratory burst and release of extracellular reactive oxygen species (ROS) which are quantified by CL. ROS are known to be highly toxic for intra-erythrocytic malaria parasites [23] and high ROS production has been correlated with rapid clearance of *P. falciparum* parasites in Gabonese children [24].

The ADRB assay has been standardized using a reference pool of hyperimmune sera as internal positive control to assign arbitrary ADRB values (ADRB index) to each serum tested. The calculation of ADRB index permitted inter-experiments comparisons and minimized inter-assays variations related to variability of donor-dependent neutrophils activity for correlation between ADRB activity and protective status of malaria-exposed individuals.

In a previous work, ADRB activity measured in an active longitudinal follow-up of villagers from Dielmo and Ndiop [25] showed significant association with protection against clinical malaria [22]. ADRB activity of antibodies in human immune sera from Dielmo and Ndiop was also significantly correlated with levels of IgG specific for PfMSP5 [26] and PfMSP1p19 [27]. That provides arguments for the vaccine candidacy of PfMSP5 and PfMSP1p19-based malaria vaccine using ADRB assay as a functional surrogate for protection.

The ADRB assay is not limited to the use of whole merozoites but can also be used to assess ADRB activity against any crude blood-stage antigens of *Plasmodium* spp. [28,29] or any other malaria vaccine candidates under study once coated onto plates [27,30].

Nevertheless, despite these very encouraging and promising results, ADRB assay showed some limitations such as the poor integrity of antigenic pool used (aggregates of merozoites), thus not allowing their adequate quantification for better reproducibility in other laboratories. Therefore, the ADRB assay requires improvement in its methodology. Several factors or parameters had a particular influence on the CL measurement of ADRB activity of sera. In this study, the emphasis was placed on factors that enhance CL intensity and sensitivity of the assay such as the integrity of merozoites surface coat that is affected by the freezing–thawing procedure, the concentration of merozoites and pH of buffer solution.

The improvement resulted in an increased CL intensity and assay sensitivity as well as a higher reproducibility following the measurement of ADRB activity of 207 sera from individuals living in two Senegalese areas with differing malaria endemicity. Our results showed that the optimized ADRB assay is a reliable functional *in vitro* assay that can be widely used to evaluate the level of malaria immunity in endemic populations and to validate merozoite-based vaccine candidates.

## 2. Material and methods

### 2.1. Study area and sample collection

This study used and analysed sera and data collected in July 2002 during a cross-sectional prospective study with intensive follow-up from healthy individuals living in Dielmo and Ndiop, two Senegalese villages with different malaria epidemiology enrolled in a long-term observational study on the acquisition and maintenance of natural immunity to malaria [25,31,32]. At this time, in Dielmo (holoendemic area, with 200 *P. falciparum* infective bites per person per year), the malaria transmission was perennial, whereas in Ndiop (mesoendemic area, with 20 *P. falciparum* infective bites per person per year), transmission was strictly seasonal occurring from July to September [33]. The details of these study sites and the longitudinal surveys carried out have been described elsewhere [31,34–36].

This study was examined and approved by the Senegalese National Health Research Ethics Committee. The project protocol and objectives were carefully explained to the assembled villagers, and informed consent, annually renewed, was obtained individually from all subjects either by signature or by thumbprint on a voluntary consent form written in French and explained to the population in their local language (Wolof and Serere) [31,37].

Briefly, sera collected during a cross-sectional sampling in July 2002 from healthy villagers from Dielmo (97) and Ndiop (110) were selected and withdrawn from the sera biobank established as part of the Dielmo and Ndiop project. The mean age of the Dielmo and Ndiop cohorts was 26.3 [range 3.4–80] and 25.6 [range 4–76.9] years respectively; the age distribution is shown in table 1. At the time of recruitment, none of the enrolled individuals used antimalarial drugs during the four weeks prior enrolment or was symptomatic for malaria during blood sampling. Blood samples were collected by venous puncture, and sera were stored at −20°C. Active clinical surveillance was done over a 5.5-month period encompassing the malaria transmission season, from August to December 2002, as described elsewhere [31].

**Table 1.** Demographic of the individuals from Dielmo and Ndiop tested for antibody-dependant respiratory burst responses against merozoites.

	DIELMO		NDIOP	
	number	mean age (years)	number	mean age (years)
effective	97	26.3	110	25.6
age groups (years)				
3–6	16	4.2	33	8.7
7–14	20	10.7	39	20.3
≥15	21	37.0	38	45.2

### 2.2. Reagents and buffers

The CL substrate was the isoluminol stock solution prepared by dissolving 4 mg of Isoluminol (4-aminophtalhydrazide, Sigma, St Louis, MO, USA) in 1 ml of DMSO (Sigma-Aldrich, St Louis, MO, USA), protected from light with aluminium foil and stored at room temperature (RT). A fresh working solution of isoluminol was prepared on the day of the experiment at 1 : 100 dilution in sterile HBSS adjusted to pH 7.0.

Suspension solution for merozoites, PMNs and isoluminol was the Hanks' Balanced Salt Solution without calcium and magnesium (HBSS, Sigma-Aldrich, St Louis, MO, USA). HBSS buffer was filter-sterilized with a 0.22 µm filter (Millipore) before usage.

### 2.3. Serum samples

Sera from subjects of Dielmo (97) and Ndiop (110) were selected according to their long-term residency (more than 30 days) during the 5.5-month period following the July 2002 cross-sectional survey. Hyper-immune serum pools (HIS) from 30 clinically immune adult residents of Ndiop and non-immune serum (NIS) obtained commercially (Biowest, France) were designated the internal positive and negative controls respectively. All sera were stored at −20°C until use.

### 2.4. Initial procedure of ADRB assay

#### 2.4.1. Merozoite preparation

Merozoites of *P. falciparum* were isolated from *in vitro* cultures of the *Uganda Palo Alto* strain routinely maintained in continuous culture in O+ human erythrocytes as previously described [38,39]. Briefly, the culture supernatants at 5% haematocrit and 5–10% parasitaemia at merozoites stage were collected and replaced daily. The collected supernatants were centrifuged at 1000 rpm to pellet and discard residual red blood cells (RBCs), then pooled daily in a bottle and stored at 4°C until the bottle was fully filled. Pooled supernatants were then centrifuged at 1500 rpm for 10 min at ambient temperature to remove RBCs' contamination and haemozoin. Free merozoites contained in the supernatant were pelleted by centrifugation at 3000 rpm for 25 min at ambient temperature. The merozoite pellet was washed twice in HBSS at ambient temperature by centrifugation at 3000 rpm for 20 min. The merozoite pellet was finally stored at −20°C into aliquots with no additive until further processing.

#### 2.4.2. Preparation of human polymorphonuclear neutrophils

Human polymorphonuclear neutrophils (PMNs) were isolated from anonymous left over venous blood collected after routine blood tests were performed at the medical laboratory of Institut Pasteur of Dakar. Blood samples were collected into EDTA-K3 tubes, layered onto Ficoll-Histopaque (density 1077, Sigma) and centrifuged at ambient temperature for 30 min at 2000 rpm. RBCs and PMNs were harvested at the interface, and residual RBCs were removed by incubation in lysis solution (8.32 g l^−1^ ammonium chloride, 0.8 g l^−1^ sodium bicarbonate, and 0.043 g l^−1^ EDTA) for 8 min at 4°C. After centrifugation at ambient temperature for 5 min at 1500 rpm, isolated PMNs were washed twice with HBSS buffer, enumerated using Trypan Blue exclusion assay. Only cell preparations with viability greater than 90% were used for testing ADRB activity. The PMNs from 8 to 10 blood samples were pooled and resuspended in HBSS to a final concentration of 1.5 × 10^6^ viable cells ml^−1^. PMNs were used immediately after harvest and isolation usually within 2–3 h following sample collection.

#### 2.4.3. ADRB assay procedure

ADRB activity was evaluated in white opaque 96-well plates by isoluminol-enhanced CL, as measured by an automated LB96 V Microlumat Plus luminometer (Berthold, Germany) controlled with WinGlow software. Briefly, 40 µl of merozoite were opsonized with 10 µl of serum sample or control sera. Plates were covered with aluminium foil and incubated for a minimum of 30 min at 37°C. After incubation, 100 µl of PMNs suspension at 1.5 × 10^6^ cells ml^−1^ were added to trigger respiratory burst. The CL detection of extracellular ROS production was initiated by a rapid addition of 100 µl of isoluminol. Plate reading started immediately after addition of isoluminol and each well was monitored automatically by the luminometer for 0.5 to 1 s at 1 min intervals for 30 min to 60 min. All measurements were carried out at a constant temperature of 37°C.

Results are expressed as relative light units per second (RLU s^−1^). A maximum peak of CL signal (RLUmax) of the curve is generally observed within minutes of initiating the reactions (around 0 to 2 min).

Results are presented as a standardized activity index of ADRB (ADRB index) according to the formula: ADRB index = (RLUmax (serum sample)/RLUmax HIS) × 1000 where RLUmax HIS is the average of duplicate measurements of the standard positive control deposited at the plate's beginning and end in each assay.

Only experiments with RLUmax of HIS greater than or equal to 100 (greater than 10× background) were included in the analyses.

Control wells in each assay consisted to (i) a blank (well with merozoite only and without serum) that provided the background signal of the instrument and any potential signal caused by the presence of impurities in the reagents, (ii) a negative control NIS (merozoites opsonized with NIS) and (iii) the positive control HIS (merozoites opsonized with hyperimmune sera) in duplicates, at the beginning and at the end of each plate.

The specificity of the ADRB assay was assessed by verifying that the CL signal intensity measured corresponds to the release of extracellular ROS by neutrophils activated by opsonized merozoites (no peak of CL signal with negative control).

The sensitivity or signal strength (signal-to-noise ratio) of ADRB assay is defined by the ratio of the specific signal generated to the background noise of the system (signal-to-noise = RLUmax HIS/RLUmax blank ≥ 4 considered optimal for use in the ADRB assay).

Experiments were performed three times in three consecutive days by the same operator in the same laboratory, with the same method, test items and equipment.

### 2.5. Statistical analysis

CL data were collected by WinGlow software and kinetic profiles for the ADRB assay were plotted using Microsoft Excel. To compare the differences in ADRB activity between two groups, Wilcoxon test and Mann–Whitney test were used. Comparisons among three or more groups were done by Kruskal–Wallis test. Another method of calculation of ADRB index using the area under the curve (ADRB_AUC_) was estimated by trapezoidal rule using Microsoft Excel. One-way ANOVA was used to compare the means of ADRB indexes calculated with both methods.

The relationship between ADRB responses and the risk of malaria attacks occurring during the 5.5 months following the blood sampling was tested using univariate and bivariate Poisson regression models with ADRB index as continuous variable. In the bivariate model, the effect of age was included as main explanatory covariate. The relative risk (RR) and the 95% confidence interval (95% CI) were calculated. *p*-values < 0.05 were considered significant.

Statistical analyses were carried out using GraphPad Prism 5 (GraphPad software) for univariate analyses (tests non-parametric) and R v. 3.0.1 software for Poisson regression analysis.

## 3. Results and discussion

### 3.1. Improvement of freezing procedure of merozoites

The ADRB assay requires a large supply of frozen *P. falciparum* merozoites. When merozoites were frozen at −20°C as previously described [22], aggregates of merozoites were often observed after thawing, thereby limiting their accurate quantification for a better inter-assay reproducibility. The use of an appropriate cryopreservation method to preserve the structure of the merozoite surface coat is an important parameter to measure ADRB activity. A glycerolyte solution was used as cryoprotectant. The protocol of cryopreservation with glycerolyte solution was initially used for the cryopreservation of *Plasmodium* spp [40]. Briefly, merozoites harvested from culture supernatants were resuspended in 1 ml of HBSS and glycerolyte solution at 1.5-fold was added dropwise over 5 min with continuous shaking. The merozoite–glycerolyte mixture was then aliquoted into cryovials and stored rapidly in liquid nitrogen for long-term cryopreservation. The efficiency of the cryopreservation procedure was evaluated after thawing based on the absence of merozoite aggregates, therefore enabling subsequent merozoite enumeration.

### 3.2. Improvement of procedures for thawing and recovery of merozoites

The thawing method used to meet the requirement for integrity of merozoite surface coat was the NaCl method originally used to thaw *P. falciparum* parasitized RBCs [41]. Briefly, cryovials were removed from liquid nitrogen and warmed 1 to 3 min at 37°C until completely thawed. The content was measured and transferred to a 15 ml centrifuge tube to which 0.2 vol of 12% NaCl solution (pre-warmed at 37°C) was added dropwise to the merozoites, and allowed to stay for 5 min at ambient temperature. Subsequently, 10 vol of 1.6% NaCl solution (pre-warmed at 37°C) was carefully added to the merozoites, which was then centrifuged for 20 min at 3000 rpm. The supernatant was discarded, and the merozoite pellet was washed twice in 10 ml of HBSS at 3000 rpm for 20 min. After the thawing procedure, merozoites were visualized free and intact, and could be enumerated.

**Figure 1.**
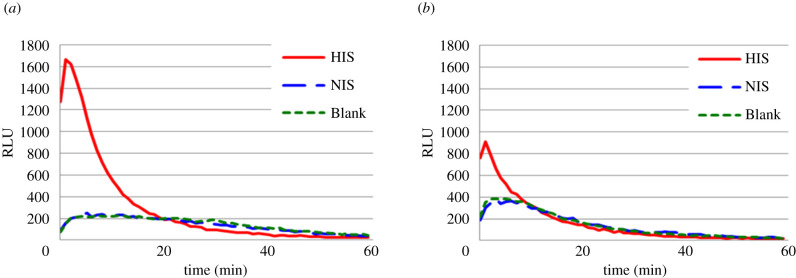
Effect of freezing method of merozoites on chemiluminescence intensity of ADRB Chemiluminescence intensity profile of neutrophils antibody-dependent respiratory burst activity performed with merozoites frozen at −20°C (*a*) and merozoites frozen in glycerolyte (*b*). 40 µl merozoites were opsonized with 10 µl standard positive control sera (HIS) and unopsonized with negative controls (blank and NIS) before activate 100 µl neutrophils (1.5 × 10^6^ cells ml^−1^).

A higher CL intensity was observed using glycerolyte-frozen merozoites (figure 1) compared to merozoites frozen at −20°C [22]. This could probably be explained by a better preservation of the integrity of merozoite surface coat due to the use of glycerolyte. The glycerolyte was subsequently used as cryoprotectant.

The integrity of the merozoite surface coat, rather than the viability of merozoites, is the essential requirement for an efficient opsonization of merozoites by specific antibodies [19–22] and the triggering of the neutrophils ADRB activity [22]. Therefore, the cryopreservation and thawing methods described above clearly provided a much better quality of merozoites for assessing ADRB activity compared to the previous study [22].

#### 3.2.1. Effect of merozoite concentration on chemiluminescence intensity of ADRB activity

The concentration of merozoites is an important parameter influencing the CL signal intensity of ADRB activity. The effect of merozoite concentration was investigated by using untreated merozoites resuspended in HBSS at 70, 60, 50, 40 and 30 × 10^6^ mz ml^−1^ as described above.

**Figure 2.**
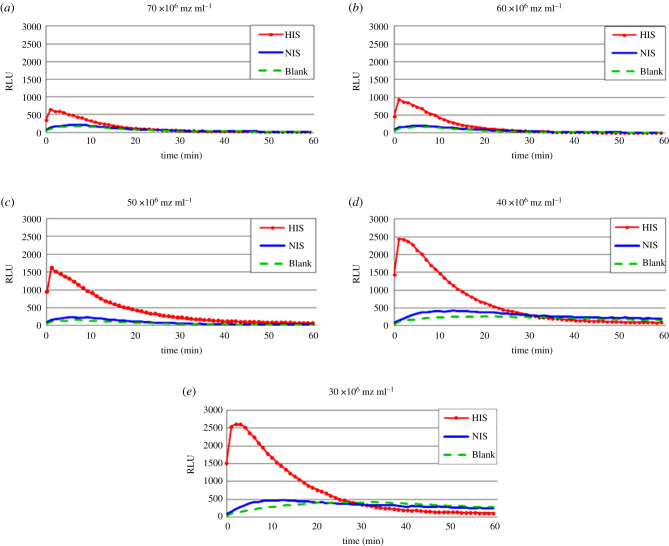
Effect of various concentrations of merozoites on chemiluminescence intensity of antibody-dependent respiratory burst activity. Chemiluminescence profiles response of various concentrations of merozoites opsonized with 10 µl of hyper-immune serum (HIS, red circle line) and unopsonized with 10 µl of non-immune serum (NIS, blue solid line) or without addition of serum (blank, green dash line) before activate 100 µl human neutrophils (1.5 × 10^6^ cells ml^−1^).

**Figure 3.**
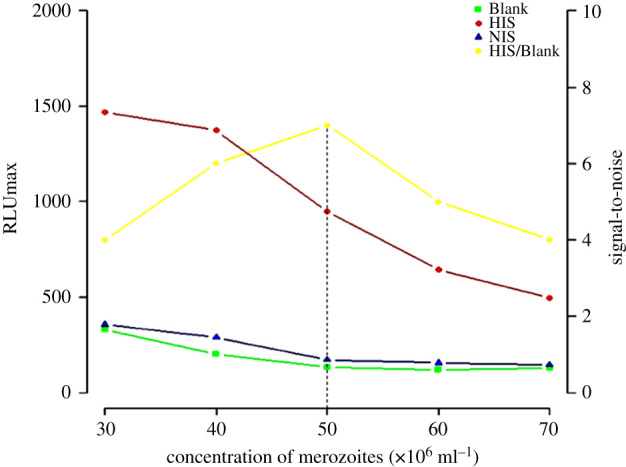
Determination of the optimal concentration of merozoite required to assess neutrophils ADRB activity. The effects of different concentrations of merozoites decreasing (70 to 30 × 10^6^ mz ml^−1^) on chemiluminescence intensity and on the signal-to-noise ratio (HIS/Blank, yellow line) were assessed at pH 7.0 and at constant temperature (37°C). Each point represents the average of RLU max and the average of signal-to-noise ratio obtained in three different ADRB experiments on three consecutive days. Best signal-to-noise ratio was obtained with the concentration of 50 × 10^6^ mz ml^−1^.

The effect of varying concentration of merozoites on CL intensity of ADRB activity is shown in figure 2. An increase of the CL intensity of HIS (positive control) and negative control (NIS, and blank) was observed proportionally to the decrease of merozoites concentration (from 70 × 10^6^ to 30 × 10^6^ mz ml^−1^). Optimal signal-to-noise output (figure 3) was obtained with concentration of 50 × 10^6^ mz ml^−1^ that was consequently selected and used for all subsequent experiments.

#### 3.2.2. Effect of increasing pH of HBSS buffer on the signal strength of ADRB activity

**Figure 4.**
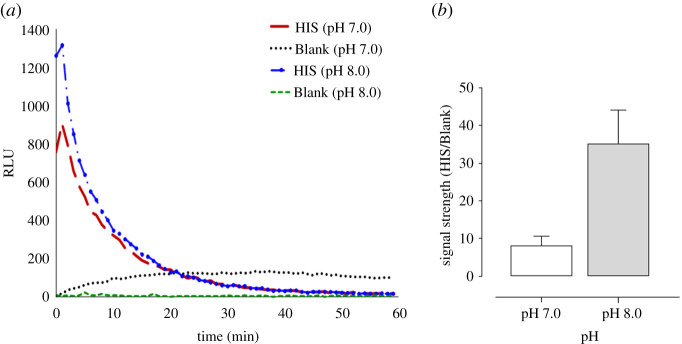
Effect of the increase of pH on chemiluminescence intensity of neutrophil antibody-dependent respiratory burst activity and on the signal strength of assay. (*a*) Chemiluminescence profiles response of neutrophil antibody-dependent respiratory burst activity (using a same PMNs pool of 1.5 × 10^6^ cells ml^−1^ and 50.10^6^ mz ml^−1^) performed at pH 7.0 and pH 8.0, in the presence of hyper-immune serum (HIS, red solid line) and non-immune serum (NIS, blue trace) and without addition of serum (blank, green trace). (*b*) Enhancement of the signal strength of ADRB assay to pH 8.0.

Since oxidation of isoluminol was found to be more efficient in an alkaline medium than in a neutral medium [42], the increase of the pH of HBSS buffer used to dilute PMNs, merozoites and isoluminol, was assessed in order to enhance the assay sensitivity. Maximum conditions for optimal isoluminol oxidation have been found between pH 8.0 and pH 12.0 [42]. Here, the pH of HBSS buffer was only adjusted to pH 7.0 and pH 8.0 for evaluating the effect of pH on CL intensity of ADRB and on the signal strength (signal-to-noise ratio). Using 1.5 × 10^6^ PMNs ml^−1^ and 50 × 10^6^ mz ml^−1^, the CL intensity (figure 4*a*) and the signal strength (figure 4*b*) were found higher at pH 8.0 than pH 7.0. Thus, HBSS buffer at pH 8.0 was selected for subsequent experiments in this study.

#### 3.2.3. Alternative calculation method of ADRB index using the area under the curve

An alternative method for calculating ADRB index by using the area under the curve (AUC) as a quantitative analysis has been proposed. The AUC is equal to the total count of CL signal (RLU) recorded over a given interval of time, closely reflecting the entire respiratory burst detected by CL in this time interval. The area under the curve may be divided into several trapezoids and can be mathematically estimated by trapezoidal rule according to the formula AUC_Ti+1_ = ∑(RLU_i_ + RLU_i+1_)/2 × (T_i+1_ – T_i_), where RLU_i_ and RLU_i+1_ are the individual measurements of CL intensity and T_i_, T_i+1_ represent the time interval between these two measures of RLU.

$${\mathrm{ADRB}}_{\mathrm{AUC}}=\frac{{\mathrm{AUC}}_{\mathrm{serum}\;\mathrm{sample}}}{{\mathrm{AUC}}_{\mathrm{HIS}}}\times 1000.$$

Based on the maximum peak of ROS production detected by CL over different time intervals of testing, the estimated AUC with respect to the maximum peak of CL (RLUmax) was found high, and probably more accurate, using the shortest test interval allowing reasonably good precision of measurement [43]. In this study, the maximum peak CL intensity (RLUmax) of the respiratory burst generated by PMNs activated by opsonized merozoites was generally recorded in a time interval between 0 and 10 min while the time to CL returning to the baseline ranges from 20 min to 60 min. Therefore, it could be hypothesized that the maximum isoluminol oxidation by ROS released by PMNs occurs in the time interval between 0 and 10 min.

To assess whether the calculation method of ADRB index using the AUC was different or equivalent to the usual method that uses the maximum peak CL value, an ADRB assay was performed with 89 sera from Ndiop and 89 sera from Dielmo with the optimized conditions (50 × 10^6^ mz ml^−1^, 1.5 × 10^6^ PMNs ml^−1^ and HBSS pH 8). The estimation of ADRB index of sera using the AUC (ADRB_AUC_) was assessed in three time intervals of CL (0–60 min, 0–20 min and 0–10 min). The ADRB index of sera from Dielmo and Ndiop calculated with the usual method was compared respectively with ADRB_AUC(0–60 min)_, ADRB_AUC(0–20 min)_ and ADRB_AUC(0–10 min)_.

A Kruskal–Wallis test showed no statistically significant difference between ADRB and ADRB_AUC(0–10 min)_, ADRB_AUC(0–20 min)_ or ADRB_AUC(0–60 min)_ (*p* = 0.9 and *p* = 0.3 for sera from Dielmo and Ndiop, respectively).

This suggests that calculating ADRB by the usual method or by the area under curve is equivalent, regardless of the time interval of CL.

#### 3.2.4. Evaluation of the optimized ADRB assay

**Figure 5.**
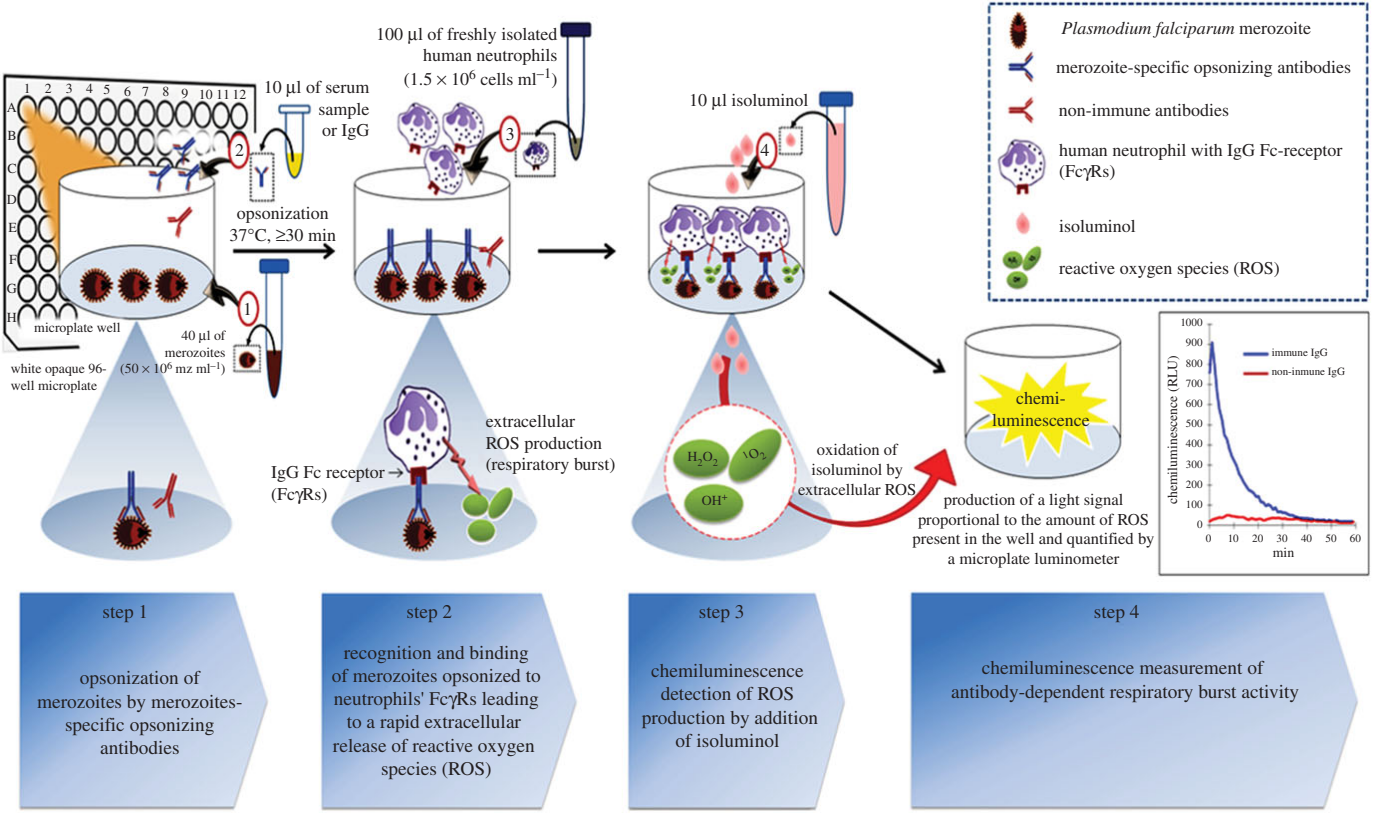
Schematic representation of the antibody-dependent respiratory burst assay procedure optimized.

Using the optimized assay conditions, the association between ADRB activity and protection against clinical malaria was assessed by analysing sera from 97 individuals (3.4 to 80 years old) from Dielmo (holoendemic area) and 110 individuals (4 to 76.9 years old) from Ndiop (mesoendemic area). The improved ADRB assay procedure is summarized in figure 5.

Briefly, 40 µl of merozoite pellet (50 × 10^6^ mz ml^−1^) was incubated with 10 µl of test or control sera in white opaque 96-well plates for at least 30 min at 37°C. Immediately following addition of 100 µl PMNs (1.5 × 10^6^ PMNs ml^−1^) and 100 µl of isoluminol (0.04 mg ml^−1^ in sterile HBSS adjusted to pH 8.0), plate reading started and continued for 60 min. Data are presented as a standardized activity index of ADRB calculated as described in the Material and methods section.

#### 3.2.5. Relationship between ADRB index and *P. falciparum* malaria endemicity

**Figure 6.**
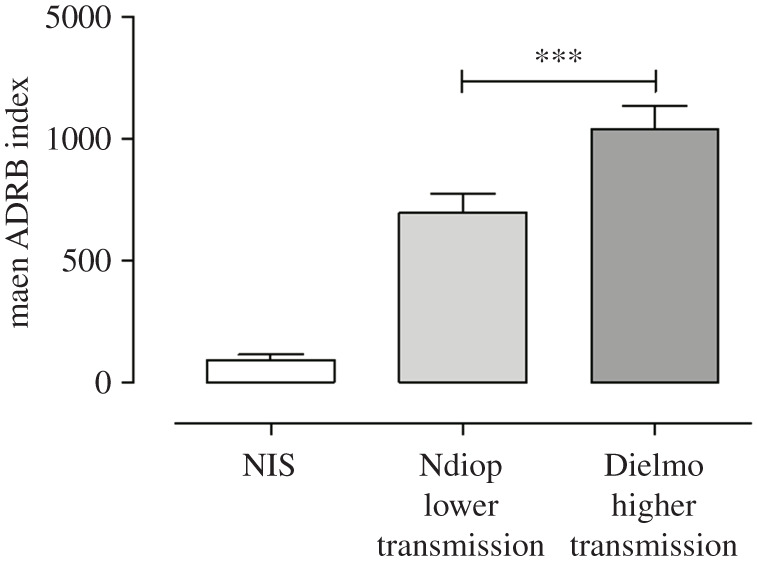
ADRB activity as a function of the endemic level of malaria. ADRB indexes of sera from villagers living in a holoendemic area (Dielmo, *n* = 97) and in a mesoendemic area (Ndiop, *n* = 110) were calculated as described in the Material and methods section using HIS as a positive internal standard in presence of negative controls (NIS). The statistics for difference of mean ADRB responses between sera from high (Dielmo) and low (Ndiop) malaria transmission are given: *p* < 0.0001, Mann–Whitney test. The significance was determined at *p* < 0.05.

The mean values of ADRB activity were significantly higher with sera from Dielmo (holoendemic) than Ndiop (mesoendemic) villagers (mean ADRB = 1041 and 697 in Dielmo and Ndiop respectively; Mann–Whitney test, *p* < 0.0001) (figure 6). This suggests that the acquisition of merozoite-specific opsonizing antibodies able to induce the neutrophils respiratory burst activity relates to the level of malaria endemicity [22].

Interventions that reduce malaria burden by limiting exposure to *P. falciparum* lead to a significant reduction of levels of antibodies inducing neutrophil ADRB activity in malaria-endemic populations. Therefore, assessing ADRB activity of sera could represent a valuable serological tool to monitor and evaluate changes in malaria exposure for efficient control strategies, and such an assumption should be investigated.

#### 3.2.6. ADRB activity in relation to age

Ages of individuals whose sera were tested in the study were categorized into three different age groups based on parasitological and clinical data gathered during the first approximately 10 years of the longitudinal study of the Dielmo–Ndiop project: 3–6, 7–15, and greater than or equal to 15 years for Dielmo, and 4–14, 15–30, and greater than or equal to 30 years for Ndiop [25,31,34,44].

**Figure 7.**
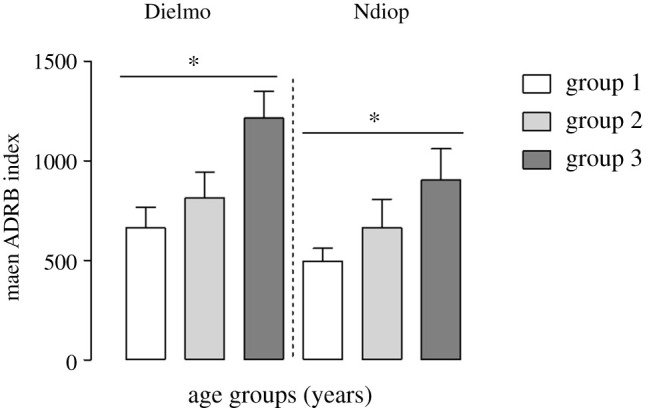
ADRB activity as a function of age group. Mean ADRB indexes of sera from Dielmo and Ndiop by age groups. Age of villagers was categorized according to parasitological and clinical data gathered throughout the longitudinal study of the Dielmo–Ndiop project into three age groups: 3–6, 7–15, and greater than or equal to 15 years for Dielmo, and 4–14, 15–30, and greater than or equal to 30 years for Ndiop. The mean of ADRB increased with age (*p* = 0.01 and 0.04 respectively for Dielmo and Ndiop). The non-parametric Kruskal–Wallis test was used for statistical analysis with a significance determined at *p* < 0.05.

The ADRB responses of sera against merozoites in the different age groups in Dielmo and Ndiop are shown in table 1. In both populations, a significant age-dependent increase of ADRB activity was observed (*p* = 0.018 in Dielmo and *p* = 0.046 in Ndiop, Kruskall–Wallis test) (figure 7). ADRB activity was highest with sera from the oldest age group (mean ADRB = 1214 and 905 respectively in Dielmo and Ndiop) and decreased progressively in the younger age group to mean ADRB of 664 and 497 in Dielmo and Ndiop, respectively.

Age (which reflects cumulative malaria exposure) is considered one of the most important factors involved in the acquisition of immunity against *P. falciparum* malaria [32]. The acquisition of opsonizing antibodies against merozoites able to induce measured ADRB activity clearly reflects the age-dependent exposure to malaria infection as reported in other studies [20–22]. This confirms that ADRB activity increases with repeated malaria exposure as previously reported [22].

The cumulative effect of malaria exposure on ADRB activity was also observed experimentally in a mouse model in which *Plasmodium yoelii* malaria infection was largely controlled by FcR-independent activity [45]. Functional anti-merozoite ADRB activity was not detectable in the *P. yoelii* model after a nonlethal primary parasite exposure, but following the induction of a second malaria infection ADRB activity was enhanced [28], thus adding weight to the previous observation that ADRB activity to *P. falciparum* increases with repeated natural exposure [22].

#### 3.2.7. ADRB activity in relation to clinical malaria episodes during the follow-up period

During the active longitudinal monitoring of clinical malaria episodes carried out in both sites during the 5.5 months following the cross-sectional blood sampling (mid-July to end of December 2002), 54 and 90 confirmed clinical malaria attacks were recorded in Dielmo and Ndiop, respectively. The association between the ADRB activity of individuals' sera and the occurrence of clinical malaria attacks during the follow-up period was investigated. The Dielmo and Ndiop cohorts were stratified into two groups according to the occurrence or not of malaria attacks: ‘no malaria episode’ (74 in Dielmo and 23 in Ndiop) and ‘malaria episodes' comprising individuals who experienced one or more malaria attacks (23 in Dielmo and 43 in Ndiop).

**Figure 8.**
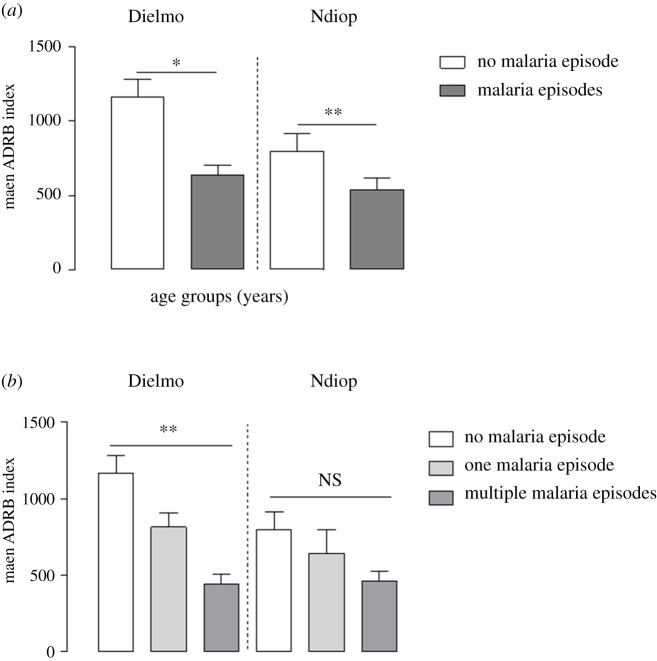
ADRB activity as function of occurrence of malaria episodes during the follow-up period (*a*) Mean ADRB indexes of sera from Dielmo (*n* = 97) and Ndiop (*n* = 110) in individuals group with no malaria episode and in those who experienced at least one malaria episode. In both study areas, the mean ADRB indexes were significantly higher in the ‘no malaria episode’ group compared with the ‘malaria episodes’ group. The non-parametric Wilcoxon test was used for statistical analysis with a significance determined at *p* < 0.05. (*b*) Mean ADRB indexes of sera from Dielmo and Ndiop for each group of malaria attacks episodes subdivided according to the number of malaria attacks into: ‘no malaria episode’, ‘one malaria episode’ and ‘multiple malaria episodes’. There was no significant difference in the protective association between the three groups in Ndiop. Statistically significant differences were determined by the non-parametric Kruskal–Wallis test. The significance was determined at *p* < 0.05.

In both study areas, ADRB activity were significantly higher in the ‘no malaria episode’ group than the ‘malaria episodes' group (mean ADRB = 1166 versus 638, *p* = 0.01, in Dielmo; mean ADRB = 798.6 versus 539.5, *p* = 0.003, in Ndiop; Wilcoxon test) (figure 8*a*).

To investigate the association between the ADRB activity and the number of malaria episodes that occurred during the follow-up period, the individuals in the ‘malaria episodes’ group was subdivided in two subgroups: ‘one malaria episode’ and ‘multiple malaria episodes’.

In the high endemic area of Dielmo, ADRB activity was highest in individuals without malaria episode and decreased significantly with the number of malaria attacks, with the lowest ADRB activity found in individuals who have had multiple malaria episodes (figure 8*b*). Mean ADRB values were 1166, 816.1 and 443.7 for the ‘no malaria episode’, ‘one malaria episode’ and ‘multiple malaria episodes’ groups, respectively (*p* = 0.001, Kruskal–Wallis test) (figure 8*b*).

A similar trend of decreasing mean ADRB activities with the number of malaria attacks was also observed in Ndiop, but without significant difference (*p* = 0.8, Kruskal–Wallis test) between the three groups (figure 8*b*). Mean ADRB values were 798, 644.2 and 464.1 for ‘no malaria episode’, ‘one malaria episode’ and ‘multiple malaria episodes’ groups, respectively (figure 8*b*).

Taken together, ADRB activity was found to be lower in individuals with multiple malaria episodes, suggesting that initial levels of anti-merozoite antibodies that trigger neutrophils respiratory burst could be predictive of immune status. Similar functional studies of antibody-mediated merozoite opsonization in children reported also reduced levels in children susceptible to multiple clinical episodes [20,21]. This suggests that the evaluation of opsonic antibodies mediating the killing activity of merozoites has a potential predictor value for immune status in children as well as adults.

This discrimination between individuals without malaria attack and those who experienced malaria episodes highlights a very important aspect of ADRB assay as a major asset for *in vitro* functional correlate of acquired immunity to malaria. A low ADRB activity appears to be a predictable factor of susceptibility to malaria episodes especially in areas of high malaria endemicity.

#### 3.2.8. Association between ADRB activity and the incidence of clinical malaria episodes

Univariate Poisson regression analysis showed that ADRB activity is significantly associated with a reduced risk to clinical malaria in Dielmo (RR < 1, 95% CI, 0.997 to 0.998, *p* < 0.0001) (table 2) and in Ndiop (RR < 1, 95% CI, 0.997 to 0.999, *p* = 0.024) (table 3).

**Table 2.** Poisson regression analysis of ADRB index values and incidence of clinical malaria in Dielmo.

variables	categories	RR [IC (95%)]	unadjusted	adjusted
			*p*-value	*p*-value
ADRB index	continuous	0.997 [0.997–0.998]		<0.001
ADRB index	continuous	0.998 [0.997; 0.999]	0.011	0.002
age (years)	≥15^a^	1		
	7.14	3.36 [1.071–11.386]	0.04	<0.001
	<7	22.35 [89.638–65.062]	<0.001	

^a^Reference category Univariate and bivariate Poisson regression models analysing the relationship between ADRB index included as continuous variable and incidence of clinical malaria occurred during the 5.5 months following the sampling in Dielmo. The bivariate model includes age groups (adults aged greater than or equal to 15 years as reference group) as categorical covariate. The relative risk (RR) was calculated and complemented by a 95% confidence interval (95% CI) and *p*-value was considered significant at *p* < 0.05.

**Table 3.** Poisson regression analysis of ADRB index values and incidence of clinical malaria in Ndiop.

variables	categories	RR [IC (95%)]	unadjusted	adjusted
			*p*-value	*p*-value
ADRB index	continuous	0.999 [0.993; 0.999]		0.02
ADRB index	continuous	0.999 [0.999; 1.00]	0.684	NS
age (years)	≥30^a^	1		
	15–29	8.10 [1.86–35.30]	0.005	<0.001
	3–14	38.05 [9.26–156.37]	<0.001	

^a^Reference category. NS: non-significant. Univariate and bivariate Poisson regression models analysing the relationship between ADRB index included as continuous variable and incidence of clinical malaria occurred during the 5.5 months following the sampling in Ndiop. The bivariate model includes age groups (adults aged ≥30 years as reference group) as categorical covariate. The relative risk (RR) was calculated and complemented by a 95% confidence interval (95% CI) and *p*-value was considered significant at *p* < 0.05.

The incidence of clinical malaria was further analysed in a bivariate Poisson regression model including the ADRB responses as continuous variable and age groups as categorical covariate (adults aged greater than or equal to 15 years and greater than or equal to 30 years in Dielmo and Ndiop respectively, as reference group).

The data reported in table 2 show that the ADRB activity was significantly associated with a reduced risk of malaria attack in all age groups in Dielmo: RR < 1; 95% CI, 0.997 to 0.999; *p* = 0.002 ([less than 7 years] versus [greater than or equal to 15 years]: RR, 22.35; 95% CI, 89.638 to 65.062; *p* < 0.001; [7–14] versus [greater than or equal to 15 years]: RR, 3.36; 95% CI, 1.071 to 11.386; *p* = 0.04).

However, in Ndiop, no significant correlation was found with protection against clinical malaria when analysis was adjusted for age group (table 3), contrasting to a previous report [22]. This difference may be due to our change of ADRB procedure. It may also suggest that in Ndiop the acquisition of immunity against malaria was age-dependent.

The association between ADRB activity and clinical protection from malaria was stronger in Dielmo, an area of intense malaria transmission with 200 infectious bites per person per year [37], where most individuals who were highly exposed to mosquito bites and did not have clinical malaria episodes during the follow-up period could be effectively protected. In contrary, in Ndiop the age-adjusted association between ADRB activity and protection was less clear. In this area, transmission was substantially low during 2002, with only 20 infective bites per person per year compared to previous and following years (greater than 50 infective bites per person per year) [37]. The lack of clinical malaria episodes in some individuals during the follow-up period could be due to a lower level of exposure to infectious mosquito bites [46].

Taken together, the findings from this study confirm the results from recent studies [29,30] that identified ADRB activity as a relevant correlate of clinical protection against malaria. This study confirms that ADRB activity is a valuable measurement for assessing the level of acquired immunity during monitoring of populations by malaria control programmes or clinical trials of malaria vaccines. However, careful attention to experimental parameters is required to ensure better reproducibility of the assay. The ability of opsonizing antibodies against merozoites to induce neutrophil ADRB activity might be an important aspect of the mechanisms that potentially contribute to protective immunity, and deserves to be taken into account. From a practical point of view, the fact that regularly obtaining access to large numbers of freshly obtained neutrophils may be an obstacle for many laboratories, and may constitute a challenge that merits attention. Further studies to explore and confirm the effectiveness of this functional assay as a correlate of protection should be considered.

## Data Availability

All relevant data are within the paper.
